# Neuroticism, Internalizing Psychopathology, and Affective Reactions to Thought Content in Daily Life

**DOI:** 10.1111/jopy.70026

**Published:** 2025-10-20

**Authors:** Henry R. Cowan, Aidan G. C. Wright, Sarah L. Pedersen, Dahlia Mukherjee, Sophie Lazarus, Jay C. Fournier

**Affiliations:** ^1^ Psychology Michigan State University East Lansing Michigan USA; ^2^ Psychiatry and Behavioral Health The Ohio State University Columbus Ohio USA; ^3^ Psychology University of Michigan Ann Arbor Michigan USA; ^4^ Psychiatry University of Pittsburgh Pittsburgh Pennsylvania USA; ^5^ Psychiatry and Behavioral Health Penn State College of Medicine, Pennsylvania State University Hershey Pennsylvania USA

**Keywords:** daily diary, ecological momentary assessment, emotion, internalizing psychopathology, mindfulness, neuroticism, rumination, thought content, within person

## Abstract

**Introduction:**

This study examined affective reactions to thought content (TC) in daily life and the influence of neuroticism and internalizing symptoms.

**Methods:**

Community young adults (*N* = 119; *n* = 80 with elevated depression/anxiety) completed assessments of neuroticism, internalizing symptoms, and daily diary measures of TC and positive/negative affect for seven consecutive days (*N* = 758 observations). Multilevel confirmatory factor analysis (MCFA) examined the structure of TC. Multilevel models examined TC‐affect relationships and moderation by neuroticism and internalizing symptoms.

**Results:**

MCFA found two TC factors: internal‐past (problems, emotions, the past) and external‐present (external events, others, the present). Internal‐past TC uniquely related to lower positive and higher negative affect within and between persons. External‐present TC uniquely related to higher positive affect within and between persons. Neuroticism and internalizing related to higher negative and lower positive affect, but neither variable significantly moderated TC‐affect relationships. Neuroticism had incremental effects over and above internalizing. At the facet level, self‐consciousness uniquely predicted lower positive affect, and angry hostility uniquely predicted higher negative affect.

**Discussion:**

TC meaningfully relates to emotion in daily life. Neuroticism and internalizing predicted overall experience of positive/negative emotion, not affective reactions to specific TC. Within‐persons, neuroticism and internalizing were differentiated by the incremental effect of neuroticism and the specific effects of neuroticism facets. Theoretical, methodological, and clinical implications are discussed.

## 1 Introduction

Neuroticism—a broad personality trait capturing emotional negativity, volatility, and reactivity (Hisler et al. 2020)—is closely entwined with internalizing psychopathology such as anxiety, depression, and posttraumatic stress. Measures of neuroticism and internalizing psychopathology have similar item content (Ormel et al. 2004); pervasively co‐occur in affected individuals (Kotov et al. 2010); and share genetic risk factors (Hettema et al. 2006) and neuroanatomical features (Hyatt et al. 2019). These similarities have fueled longstanding debates about distinctions between neuroticism and internalizing psychopathology (e.g., Barlow et al. 2014; Griffith et al. 2010). As Ormel and colleagues have argued, “even prospective associations of neuroticism with mental health outcomes are basically futile, and largely tautological” because neuroticism simply measures a stable, trait‐like component of psychological distress (Ormel et al. 2004, p.906). Reviewing large‐scale studies on the latent structure of personality and psychopathology, Watson and colleagues similarly concluded that neuroticism “represents the central feature of internalizing. Indeed, cross‐sectional data show that individual differences in broadly conceptualized internalizing psychopathology and [neuroticism] are very highly correlated and essentially fungible” (Watson et al. 2022, p.31).

Between‐person correlations between neuroticism and internalizing symptoms can, however, be explained by at least seven different theoretical and causal models (Klein et al. 2011). These models make different predictions about etiology, comorbidity, risk, and treatment that can be observed at the within‐person level, by studying variation in intraindividual processes over time. For instance, longitudinal studies supporting the vulnerability model have shown that elevated neuroticism tends to precede the development of internalizing symptoms and diagnoses within persons (Aldinger et al. 2014; Ormel et al. 2013; Williams et al. 2020, 2021). Other designs focus on the study of within‐person variation. Traits are, by definition, relatively stable, yet internalizing symptoms and psychological distress fluctuate (Watson et al. 2022). For any given person, the presence of elevated trait neuroticism does not guarantee the presence of internalizing symptoms or distress on any given day. Rather, intermediate mechanisms must connect neuroticism and its facets to symptom expression and distress in daily life. These mechanisms can be studied through within‐person experience sampling and daily diary methods, which capture the dynamic interplay between thoughts and emotions in daily life. This approach has found that lower positive affect, higher negative affect, and higher affective reactivity are associated with neuroticism (e.g., Bolger and Schilling 1991; Hisler et al. 2020; Ringwald et al. 2024) and internalizing symptoms in daily life (e.g., Mukherjee et al. 2023; Naragon‐Gainey 2019; Zhu et al. 2022). It is unclear to what extent these studies reflect shared versus unique variance across neuroticism and internalizing symptoms.

These processes are important targets because mechanisms of change in neuroticism and internalizing are not fully understood and appear not to be fully overlapping. Neuroticism is normally distributed in clinical internalizing samples, and neuroticism scores predict treatment response in these samples (Fournier et al. 2020, 2019). This suggests that meaningful components of neuroticism cannot be accounted for simply by psychological distress, and that these components may operate differently within individuals over time. Importantly, psychiatric interventions appear to have differential effects on neuroticism and internalizing symptoms. Psychotherapy has larger effect sizes for changes in depression or anxiety symptoms (Bandelow et al. 2015; Barlow et al. 2017; Munder et al. 2019) than for changes in neuroticism (Roberts et al. 2017; Sauer‐Zavala et al. 2021). By contrast, one randomized controlled trial of paroxetine (an antidepressant medication) found that paroxetine had a larger specific effect relative to placebo on neuroticism than on depression symptoms (Tang et al. 2009). Notably, both psychotherapy (Sauer‐Zavala et al. 2021) and paroxetine (Tang et al. 2009) led to lower neuroticism scores after controlling for improvement in depression/anxiety symptoms. Taken together, this evidence suggests that neuroticism and internalizing symptoms may change by somewhat different mechanisms. For this reason, it is important to define shared and unique mechanisms of neuroticism and internalizing in daily life to work toward understanding differences in mechanisms of change.

In sum, the study of within‐person processes has theoretical implications for disentangling neuroticism from internalizing symptoms, by identifying processes that are fungible and processes that are unique. This approach has important practical implications for understanding mechanisms of change that can inform treatment selection (e.g., choosing a transdiagnostic treatment vs. a disorder‐specific treatment; Sauer‐Zavala et al. 2021).

### 1.1 Affective Reactions to Thought Content

Affective reactions to thought content may distinguish neuroticism from internalizing psychopathology at the within‐person level. Whereas neuroticism is typically understood as a general tendency toward aversive emotions, social‐cognitive models of depression and anxiety posit that maladaptive cognitive styles (e.g., core schemas) prompt specific kinds of thought content in daily life (e.g., about one's helplessness, worthlessness, or unlovability; Beck 1979) that produce aversive emotions (Abramson et al. 2002; Andrews‐Hanna et al. 2013; Beck 1979). Supporting this theory, one study compared a group of depressed individuals to a group of non‐depressed peers over a two‐week period, finding that depressed individuals' daily thought content (descriptions of naturally occurring events) contained more negative and less positive emotional language, and more pronouns referring to the self and others (Krejtz et al. 2020). Neuroticism may have similar effects: previous studies have found that neuroticism interacts with negative interpretations of daily events to predict depressive symptoms (Hankin et al. 2005) and generation of additional stressful events (Hankin 2010). Neuroticism and internalizing may have shared and unique effects on affective reactions to different kinds of thought content in daily life. Affective reactions may differ in response to, for example, internally‐ versus externally‐directed cognition (Dixon et al. 2014) or past‐ versus present‐ versus future‐focus (McKay et al. 2017).

### 1.2 Neuroticism Facets

Additionally, differential effects of neuroticism and internalizing symptoms may be clearer at the level of specific facets rather than the broad trait of neuroticism. At the within‐person level, prospective links have been reported between specific neuroticism facets and specific internalizing syndromes (Williams et al. 2020; Zinbarg et al. 2016). Notably, a recent study (Fournier et al. 2019) found that the neuroticism facets of self‐consciousness (which captures tendencies toward self‐focused attention and self‐conscious emotions) and angry hostility (which captures tendencies toward aggression) were independently associated with different types of difficulties in interpersonal functioning over and above a general negative affectivity factor. This suggests that these components of neuroticism are clinically meaningful and distinct, even after accounting for the effect of psychological distress. Importantly, these facets do not recapitulate the item content of anxiety and depression symptoms (e.g., compared to the neuroticism facets of “depression” and “anxiety”). Fournier et al. (2019) tested these facets' effects on interpersonal functioning, and additional research is needed to determine: (a) their effects on intrapersonal functioning; and (b) whether other facets might be relevant for intrapersonal functioning.

### 1.3 The Present Study

In a group of participants oversampled for internalizing symptoms (67% with at least mild symptoms of depression or anxiety), the present study examined relationships between thought content and positive/negative affect in daily life and tested moderation by neuroticism and internalizing symptoms. Relationships between daily thoughts and daily affective experience were defined in mixed‐effects models of daily diary data. Interactions were examined to determine whether neuroticism or internalizing symptoms were associated with different affective reactions to thought content. We hypothesized that neuroticism and internalizing symptoms would relate to higher levels of negative affect and lower levels of positive affect (main effects) and accentuate the effects of thought content on increasing negative affect and decreasing positive affect (cross‐level interactions with thought content). Neuroticism and internalizing symptoms were also tested head‐to‐head in the same models to determine which effects were shared or unique. Theoretically distinct and empirically relevant neuroticism facets (self‐consciousness and angry hostility, see Fournier et al. 2019) were examined in secondary analyses to test for unique effects on daily cognitive‐affective experience after accounting for the effect of internalizing symptoms. Finally, in exploratory analyses, the effects of all six neuroticism facets were tested separately.

## 2 Material and Methods

### 2.1 Participants

Participants were 119 young adults aged 18–25 years recruited from the community, oversampled for internalizing psychopathology (*n* = 80 [67%] had at least mild symptoms of depression or anxiety, defined as QIDS ≥ 6 or PROMIS‐A ≥ 55). No participants were currently receiving psychiatric medication or psychotherapy, and no treatment was provided as part of this study or delayed for participation. Exclusion criteria included a lifetime diagnosis of bipolar disorder or a psychotic disorder, current substance use disorder, lack of English proficiency to complete study procedures, estimated premorbid IQ < 85, visual disturbance, as well as exclusion criteria common for the neuroimaging components of the study beyond the scope of this manuscript (left handedness, failure of standard fMRI screen, history of head injury, current pregnancy, developmental disorder, or systematic medical disease). Numbers of excluded participants and exclusion reasons are shown in Supporting Information S4 (Table S4). Participants' demographic, clinical, and personality characteristics are shown in Table 1.

**TABLE 1:** Descriptive statistics: sample characteristics and between‐person variables.

Variable	*N* or *m*	% or SD	Skew	Kurtosis	Between‐person correlations				
					Internalizing	Neuroticism	Ang‐Host	Self‐Cons	Pos Aff
*N*	119								
Age	21.94	2.87	1.02	0.87					
Sex assigned at birth									
Female	81	68.1	—	—					
Male	38	31.9	—	—					
Race									
Asian	19	16.0	—	—					
Black	8	6.7	—	—					
White	80	67.2	—	—					
Other/multiple	12	10.1	—	—					
Ethnicity									
Hispanic/Latino	9	7.6	—	—					
Education									
Some high school	1	0.8	—	—					
High school diploma	21	17.7	—	—					
Some college	50	42.0	—	—					
College diploma	39	32.8	—	—					
Graduate degree	8	6.7	—	—					
Symptoms									
PROMIS anxiety	20.23	7.95	−0.20	−1.15					
QIDS	8.15	5.36	0.14	−1.04					
HAMD	10.73	7.22	0.06	−0.92					
MADRS	13.82	10.59	0.07	−1.39					
Internalizing composite	0.00	1.00	−0.02	−1.13					
Personality traits/facets									
Neuroticism	100.7	35.1	−0.38	−0.54	0.79***				
Self‐consciousness	18.36	7.26	−0.11	−0.96	0.69***	0.85***			
Angry hostility	12.67	5.66	0.15	−0.55	0.63***	0.78***	0.57***		
Affect (between‐person)									
PANAS‐positive	18.34	14.52	0.99	0.41	−0.44***	−0.48***	−0.48***	−0.27*	
PANAS‐negative	37.22	17.85	0.42^a^	−0.33	0.53***	0.50***	0.35**	0.45***	−0.14

Abbreviation: TC = thought content.

^a^ Although between‐person PANAS‐Negative scores (i.e., person‐mean scores) were only somewhat skewed, daily PANAS‐Negative scores used as outcomes in multilevel models were more positively skewed (skew = 1.24) and were square‐root transformed for analysis (transformed skew = 0.16).

* False discovery rate‐corrected *p* < 0.05.

** FDR‐corrected *p* < 0.01.

*** FDR‐corrected *p* < 0.001.

### 2.2 Procedures

Participants completed a baseline assessment including demographics (age, education, racial/ethnic identity, and sex assigned at birth), self‐reported and clinician‐rated depression and anxiety, and self‐reported personality traits. Participants then completed seven consecutive days of daily diary assessments (*N* = 758 observations, range = 2–7 observations per participant, mean = 6.39, median = 7, mode = 7). Each evening, participants were prompted to sign into a secure website and complete a survey on emotions, TC, sleep, substance use, impulsivity, work performance, stress, and social/interpersonal behavior. The present study included the emotion and TC data. Participants received multiple prompts to complete the daily diary assessment over a 2‐h period beginning 30 min prior to their self‐reported bedtime. Participants were free to take as long as needed to complete the survey (due to branching logic, participants responded to a different number of items each day; completion time range = 1.9–60.7 min; mean = 8.2; median = 6.0). Study procedures were approved by the relevant Institutional Review Board, and all participants provided written informed consent.

The present study's data were collected as part of a larger project examining mechanisms of self‐reference and emotion regulation relating to internalizing symptoms and neuroticism. Sample size for the larger project was determined by a priori power analyses to identify medium‐sized effects in cross‐sectional comparisons (*d* > 0.50, *ω* ^2^ > 0.06, *f* ^2^ > 0.15) at *α* = 0.05 with 80% power. Multilevel sensitivity analyses were conducted for the present study's analyses via Monte Carlo simulation in the *mlmpower* R package (Enders et al. 2025). See Supporting Information S1 for full details. For target effect sizes (a total of *R* ^2^ = 0.13 attributable to variables at within and between person levels, a total cross‐level *R* ^2^ = 0.013 for Models 3 and 4, and a total cross‐level *R* ^2^ = 0.026 for Model 5), models were powered ≥ 0.68 to detect effects of interest. Power estimates for a range of plausible effect sizes are shown in Table S1.1. Estimated power for target effect sizes compares favorably to power traditionally achieved in psychiatric studies (median power < 0.30, Dumas‐Mallet et al. 2017).

### 2.3 Measures

#### 2.3.1 Baseline Assessment

##### 2.3.1.1 Internalizing Psychopathology

Anxiety was assessed by the Patient Reported Outcomes Measurement Information System Anxiety Short Form (PROMIS‐A; *ω*_hierarchical_ = 0.92), an 8‐item self‐report measure of general anxiety severity in the past 7 days (Pilkonis et al. 2011). Depression measures included the Hamilton Rating Scale for Depression (HAMD; *ω*_hierarchical_ = 0.77), a 17‐item clinician‐rated measure of current depression severity (Hamilton 1960); the Montgomery–Åsberg Depression Rating Scale (MADRS; *ω*_hierarchical_ = 0.83), a 10‐item clinician‐rated scale of current depression severity (Montgomery and Åsberg 1979); and the Quick Inventory of Depressive Symptoms‐Self Report (QIDS; *ω*_hierarchical_ = 0.82), a 16‐item self‐report measure of depression severity in the past 7 days (Rush et al. 2003). Internalizing measures were highly correlated and were combined into a single index of internalizing psychopathology (see “Data Analysis” section below).

##### 2.3.1.2 Personality Traits/Facets

The trait of neuroticism (*ω*_hierarchical_ = 0.84) and its constituent facets of self‐consciousness (*ω*_hierarchical_ = 0.76) and angry hostility (*ω*_hierarchical_ = 0.63) were assessed by the NEO Personality Inventory‐Revised (NEO PI‐R) (Costa and McCrae 1992), a 240‐item inventory measuring the five‐factor model of personality at the factor and facet levels.

#### 2.3.2 Daily Diary

##### 2.3.2.1 Positive and Negative Affect

Daily positive and negative affect were assessed by a reduced version of the Positive and Negative Affect Schedule—Expanded Form (PANAS‐X; Clark and Watson 1994) with a daily prompt (“To what extent did you feel the following today…”). The PANAS‐X was chosen for its coverage of multiple basic positive and negative emotions. To reduce participant burden for daily diary assessment, the 60‐item PANAS‐X was reduced to 15 items by retaining three items from each of five basic emotion scales: joviality and self‐assurance (positive); and fear, hostility, and sadness (negative). Daily positive and negative affect scores were calculated as means of positive and negative emotion items. Within‐ and between‐person reliabilities (Geldhof et al. 2014) were high for both scales: positive affect *ω*_within_ = 0.81, *ω*_between_ = 0.94; negative affect *ω*_within_ = 0.81, *ω*_between_ = 0.96.

##### 2.3.2.2 Thought Content

Daily TC was assessed by eight items rating the extent to which thoughts focused on a given type of content each day (i.e., “Throughout the day today, how much did your thoughts focus on…”). Items are presented in Table 2 below. See Supporting Information S2 for additional detail on scale construction. Each item was rated on a slider ranging from 0 = not at all to 100 = very much. Daily TC variables were defined through multilevel factor analysis, as described in “Data Analysis” section below.

### 2.4 Data Analysis

#### 2.4.1 Overall Strategy

Analyses were conducted in R version 4.3.1 (R Core Team 2018). The overall analytic strategy was to: (1) define latent variables describing TC in the daily diary data; (2) compute a series of increasingly complex nested multilevel models predicting daily positive and negative affect from TC, neuroticism, and internalizing symptoms; then (3) examine model fit comparisons and fixed‐effects estimates to determine the shared and unique effects of neuroticism and internalizing on daily cognitive–affective experience.

#### 2.4.2 Daily TC Structure

Daily TC latent variables were defined through exploratory factor analysis (EFA) and multilevel confirmatory factor analysis (MCFA). First, an initial candidate model was developed through EFA. A separate EFA was conducted for each day of daily diary data, with minimum residual factoring and oblique (oblimin) rotation. Item loadings for the initial candidate model were then averaged across the 7 days of daily diary data. The number of factors was the same for all daily EFAs and was determined by the modal number of principal components across separate parallel analyses of each day of daily diary data (parallel analysis was conducted on principal components, which have been shown to be more accurate than principal factors in parallel analysis; Lim and Jahng 2019).

After the initial candidate model was developed, MCFA was used to test whether the model fit well at both within‐ and between‐person levels. Four MCFA models were computed with: (1) 1 within‐person factor and 1 between‐person factor; (2) 1 within‐person factor and the candidate model specified as a between‐person factor; (3) the candidate model specified at the within‐person level and 1 between‐person factor; and (4) the candidate model specified at both the within‐ and between‐person levels. Model fit statistics were examined to determine adequate absolute model fit (at least 2 of CFI ≥ 0.90, RMSEA ≤ 0.06, SRMR ≤ 0.08; Hu and Bentler 1999) and best relative model fit (indicated by CFI, RMSEA, and SRMR). If no models achieved adequate absolute model fit, two respecification options were considered: (1) allowing item cross‐loadings; and (2) dropping items with low primary item loadings and/or substantial cross‐loadings. Once a final MCFA model was accepted based on meeting absolute fit criteria and achieving best relative model fit, estimated within‐ and between‐person factor scores were extracted from the CFA model for further analysis.

#### 2.4.3 Multilevel Models of Within‐Person Variation

##### 2.4.3.1 Modeling Strategy

The study's main analyses employed multilevel models predicting daily positive and negative affect from TC, neuroticism, and internalizing psychopathology. TC scores were extracted from MCFA, as described above. Between‐person TC refers to an individual's general tendency to think about certain kinds of topics. Within‐person TC refers to daily variation around those general tendencies (i.e., is the person thinking more or less about a certain kind of topic today, compared to their personal mean). Variability in within‐ and between‐person TC is orthogonal. Separating orthogonal within‐ and between‐person components is considered best practice in applications of multilevel modeling to within‐person variation (Kreft et al. 1995; West et al. 2011).

##### 2.4.3.2 Effects of Neuroticism and Internalizing

For each outcome (daily positive affect and daily negative affect), a series of progressively more complex mixed‐effects models was calculated in {lme4} v.1.1–27.1 (Bates et al. 2015) with restricted estimation maximum likelihood estimation and nloptwrap optimization. Model 1 (random slopes) included a random intercept and random slopes for affect clustered within participant ID. Model 2 (TC) added fixed effects for within‐ and between‐person TC. Models 3 (TC × neuroticism) and 4 (TC × internalizing) added a fixed main effect and interactions with within‐person TC for neuroticism (Model 3) or internalizing (Model 4). Model fit statistics and fixed‐effects estimates were examined to determine relationships between neuroticism/internalizing, daily TC, and affect.

##### 2.4.3.3 Direct Comparison of Neuroticism Versus Internalizing

Model 5 (TC × neuroticism and TC × internalizing) combined all terms in Models 3 and 4. Model fit comparisons between Model 5 and Models 3 and 4, as well as fixed effects estimates in Model 5, were examined to determine whether neuroticism or internalizing had unique effects over and above one another.

##### 2.4.3.4 Secondary Analysis of Neuroticism Facets

Model 6 tested the main effects of self‐consciousness, angry hostility, and internalizing symptoms in a single model, as these were identified as facets of interest by Fournier et al. (2019). This analysis was considered to be secondary because correlations between neuroticism facets and internalizing symptoms are likely to decrease the stability of estimated coefficients. In exploratory analyses, the effects of all six neuroticism facets were tested in separate models with the same model structure as Model 5 above, except that a neuroticism facet replaced the overall neuroticism score. Because these analyses were exploratory and not part of a nested model sequence, the significance level was set at *α* = 0.008 (0.05/6 facet models).

##### 2.4.3.5 Sensitivity Analyses

Two sets of sensitivity analyses were conducted. First, participants were identified as influential outliers based on extreme values in both Level 2 (participant‐level) residuals and influence (Cook's distance) in the combined neuroticism and internalizing model (Model 5). Four participants were identified as influential outliers (see Figures S3.1 and S3.2). Additionally, a separate group of three participants was identified with extremely high scores on internal‐past or external‐present TC (see Figures S3.3 and S3.4). This group only partially overlapped with the group of influential outliers (one overlapping participant). Therefore, separate sensitivity analyses were conducted, excluding each of these two groups of participants. Sensitivity analyses are reported in detail in the supplemental material and summarized in the main text.

## 3 Results

### 3.1 Preliminary Analyses

The PROMIS‐A, QIDS, HAMD, and MADRS were strongly correlated (*r*s ranging from 0.77 – 0.94, all *p*_FDR_ < 0.001), suggesting that they reflected a single latent internalizing dimension. Neuroticism was also strongly correlated with the four internalizing measures (*r*s ranging from 0.81 – 0.83, all *p*_FDR_ < 0.001). A single unrotated latent factor was extracted from the four internalizing measures by exploratory factor analysis. This factor accounted for 89% of the variance across internalizing scales and correlated strongly with neuroticism, *r* = 0.79, *p* < 0.001, consistent with previous findings of strong cross‐sectional associations between neuroticism and internalizing symptoms. The internalizing latent factor was used as the indicator of internalizing psychopathology in all further analyses.

### 3.2 Factor Analysis of Daily Thought Content

TC factors were derived from the daily diary TC items through EFA (to establish a candidate factor structure) and multilevel CFA (to test the candidate structure at within‐ and between‐person levels). Parallel analysis of TC items on each day indicated a mode of two factors, and two factors were interpretable in each day's factor solution. A two‐factor EFA was conducted for each day of daily diary data, with item loadings averaged across the 7 days. The resulting candidate factor structure is shown in Table 2.

**TABLE 2:** Daily Thought Content scale: item loadings from exploratory factor analysis; standardized loadings and external correlations from multilevel confirmatory factor analysis.

	EFA candidate model		MCFA within‐person		MCFA between‐person	
	Internal‐past	External‐present	Internal‐past	External‐present	Internal‐past	External‐present
“Throughout the day today, how much did your thoughts focus on…”						
Your feelings or emotions	**0.67**	0.12	0.67	—	0.76	—
Problems or challenges in your life	**0.62**	0.06	0.42	—	0.75	—
The past	**0.54**	0.08	0.41	—	0.86	—
The present	0.10	**0.33**	—	0.36	—	0.30
Acquaintances, co‐workers, or any other people in your life	0.14	**0.32**	—	0.29	—	0.67
External things (politics, sports, news events, weather, etc.)	0.04	**0.28**	—	0.34	—	0.36
The future	**0.43**	0.08	—	—	—	—
Family members, significant others, or people in your life to whom you feel close	0.26	**0.38**	—	—	—	—
Factor covariances						
Internal‐past TC	—	0.34	—	−0.05	—	0.61
Correlations						
Positive affect	—	—	−0.11**	0.13***	0.09	0.23*
Negative affect	—	—	0.34***	−0.02	0.77***	0.64***
Internalizing composite	—	—	—	—	0.31*	0.24
Neuroticism	—	—	—	—	0.26	0.20
Self‐consciousness	—	—	—	—	0.16	0.12
Angry hostility	—	—	—	—	0.31*	0.20

*Note:* “The future” and “close others” were omitted from MCFA to achieve acceptable model fit (see text for details). EFA loadings were averaged across separate EFAs conducted for each day of daily diary data. Bolding in EFA columns indicates factors to which items were assigned in MCFA. Pearson correlations reported for multilevel factor scores, estimated by Thurstone's regression‐based method.

Abbreviations: EFA = exploratory factor analysis; MCFA = multilevel confirmatory factor analysis.

* False discovery rate‐corrected *p* < 0.05.

** FDR‐corrected *p* < 0.01.

*** FDR‐corrected *p* < 0.001.

This candidate structure was then tested through MCFA. MCFA fit statistics for combinations of 1‐ and 2‐factor models at the within‐ and between‐person levels are shown in Table S2.1. The model with 2 factors at the within‐ and between‐person levels had the best relative fit but failed to meet absolute fit criteria, CFI = 0.784, RMSEA = 0.061, SRMR_within_ = 0.064, SRMR_between_ = 0.070. Two items (“the future” and “close others”) were identified as problematic in this model due to low primary loadings or modification indices suggesting cross‐loadings. Two model respecifications were attempted (see Supporting Information S2 for full results).

In respecification 1, “the future” and “close others” were allowed to cross‐load on both factors at the within‐ and between‐person level. This modification resulted in a convergence error (an element of the optimization gradient failed to approach zero). Additionally, two variants of this model were run, each of which allowed one of the two items to cross‐load. Both of these variant models converged, with the best marginal improvement in fit observed for the model allowing “the future” to cross‐load, CFI = 0.814, RMSEA = 0.058, SRMR_within_ = 0.058, SRMR_between_ = 0.067.

**FIGURE 1:**
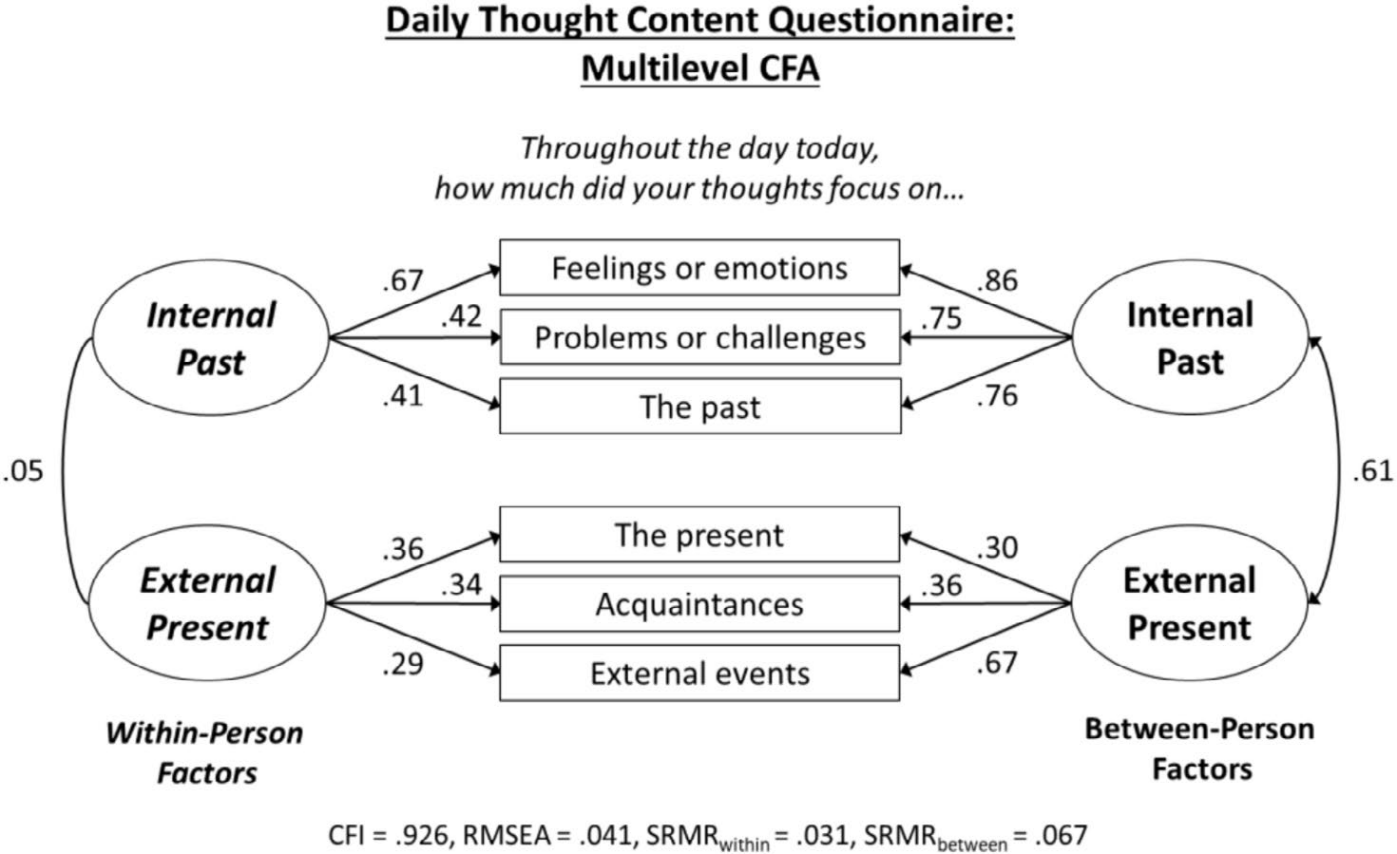
Multilevel factor structure of thought content items, defined by multilevel confirmatory factor analysis (MCFA) of 7 days of daily diary data (*n* = 119 young adults oversampled for internalizing symptoms). The thought content questionnaire also included items for thoughts about family members and the future; these items were dropped to improve MCFA model fit.

In respecification 2, “the future” and “close others” were dropped from the model. This modification markedly improved model fit and met absolute fit criteria on all fit statistics, CFI = 0.926, RMSEA = 0.041, SRMR_within_ = 0.031, SRMR_between_ = 0.067. Additionally, two variants of this model were run, each of which dropped one of the items. The model that only dropped “the future” met absolute fit criteria, CFI = 0.878, RMSEA = 0.048, SRMR_within_ = 0.050, SRMR_between_ = 0.063, but had worse relative fit than the model that dropped both items. The model that dropped “the future” and “close others” was therefore retained as the final model (see Table 2 and Figure 1). Within‐ and between‐person factor scores were estimated via Thurstone's regression method for use in primary analyses.

### 3.3 Multilevel Models

#### 3.3.1 Thought Content and Affect

##### 3.3.1.1 Positive Affect

**FIGURE 2:**
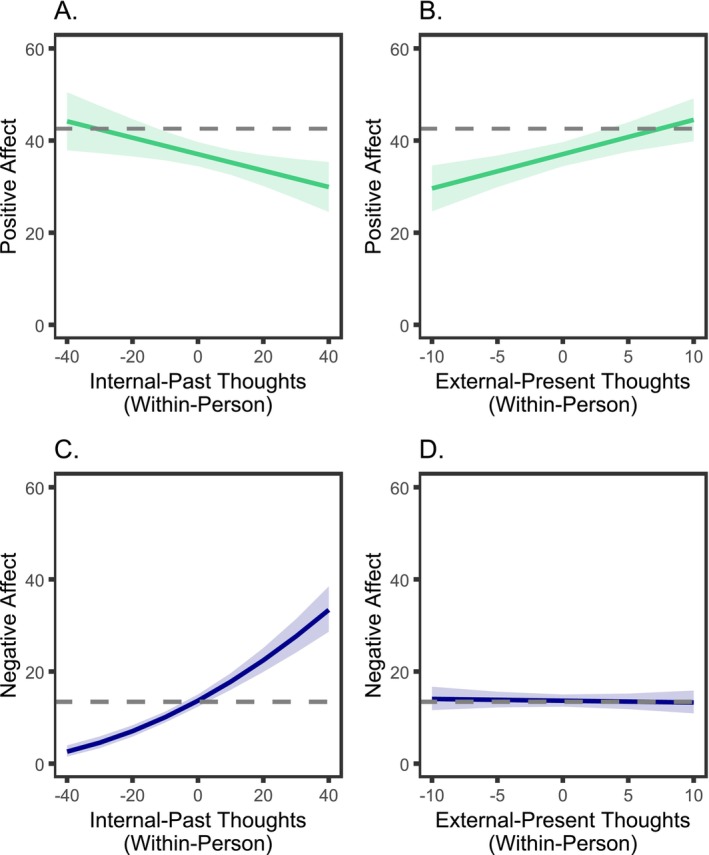
Estimated effects of thought content on daily affect within persons over 7 days of daily diary assessment (*n* = 119 young adults oversampled for internalizing symptoms). Effects were extracted from Model 5 shown in Table 4. Negative affect findings appear curvilinear because negative affect was square root transformed to correct skew prior to analyses, then back‐transformed to its original metric for plotting. Dashed lines indicate normative levels of daily affect reported in daily diary studies of 542 adults (Clark and Watson 1994; Table 5). Effects shown with 95% confidence bands.

In all models, at the between‐person level, higher levels of positive affect were experienced by individuals who reported lower neuroticism, lower internalizing symptom severity, or a tendency to experience more external‐present TC during the daily diary period. At the within‐person level, participants experienced more daily positive affect on days when they reported higher‐than‐usual external‐present TC and lower‐than‐usual internal‐past TC (see Figure 2).

##### 3.3.1.2 Negative Affect

In all models, at the between‐person level, higher levels of negative affect were experienced by individuals who reported higher neuroticism, higher internalizing symptom severity, or a tendency to experience more internal‐past TC during the daily diary period. At the within‐person level, participants experienced more daily negative affect on days when they experienced higher‐than‐usual internal‐past TC (see Figure 2).

#### 3.3.2 Neuroticism and Internalizing

Model comparisons (Table 3) indicated that Models 3 (Neuroticism) and 4 (Internalizing) predicted daily positive and negative affect better than a comparison model with affect predicted by TC alone. Models 3 and 4 (see Table 4) found similar effects for neuroticism and internalizing symptoms, both of which predicted lower positive affect and higher negative affect. No cross‐level interactions between neuroticism, internalizing, and thought content were significant.

**TABLE 3:** Fit statistics for linear mixed‐effects models predicting daily positive and negative affect.

Model	AIC	BIC	Deviance	Model comparison(s)
Models predicting daily positive affect				
1. Random Slope	6363.1	6400.1	6347.1	
2. TC	6338.6	6394.2	6314.6	vs. 1 *p* < 0.001
3. TC × Neuroticism	**6297.3**	**6366.8**	6267.3	vs. 2 *p* < 0.001
4. TC × Internalizing	6302.4	6371.8	6272.4	vs. 2 *p* < 0.001
5. TC × Neuroticism and Internalizing	6297.6	6380.9	6261.6	vs. 3 *p = 0*.123; vs. 4 *p = 0*.013
Models predicting daily negative affect				
1. Random Slope	2664.0	2701.0	2648.0	
2. TC	2496.1	2537.8	2478.1	vs. 1 *p* < 0.001
3. TC × Neuroticism	**2448.3**	**2503.9**	2424.3	vs. 2 *p* < 0.001
4. TC × Internalizing	2466.0	2521.5	2442.0	vs. 2 *p* < 0.001
5. TC × Neuroticism and Internalizing	2451.2	2520.7	2421.2	vs. 3 *p* = 0.378; vs. 4 *p* < 0.001

*Note: p*‐values based on chi‐squared tests of deviance. Negative affect was square root transformed to adjust for skew. Bold indicates lowest values of AIC and BIC.

Abbreviation: TC = thought content.

**TABLE 4:** Neuroticism, Internalizing, and Daily TC predicting daily positive and negative affect: fixed effects estimates with 95% confidence intervals.

	Daily positive affect			Daily negative affect		
	Model 3	Model 4	Model 5	Model 3	Model 4	Model 5
Level 1 (within‐person)						
Internal‐past TC	**−0.18** ** (**−0.31 to −0.04**)	**−0.18** ** (**−0.31 to −0.05**)	**−0.18** ** (**−0.31 to −0.05**)	**0.05** *** (**0.04–0.06**)	**0.05** *** (**0.04–0.06**)	**0.05** *** (**0.04–0.06**)
External‐present TC	**0.76** *** (**0.36–1.16**)	**0.77** *** (**0.37–1.17**)	**0.74** *** (**0.34–1.15**)	−0.01 (−0.04 to 0.02)	−0.01 (−0.04 to 0.02)	−0.01 (−0.03 to 0.02)
Level 2 (between‐person)						
Internal‐past TC	−0.13 (−0.44 to 0.19)	−0.10 (−0.43 to 0.22)	−0.09 (−0.40 to 0.22)	**0.07** *** (**0.04–0.09**)	**0.07** *** (**0.04–0.09**)	**0.06** *** (**0.04–0.09**)
External‐present TC	**1.01** ** (**0.40–1.62**)	**1.03** ** (**0.41–1.66**)	**1.03** ** (**0.42–1.63**)	0.02 (−0.02 to 0.06)	0.02 (−0.02 to 0.07)	0.02 (−0.02 to 0.06)
Neuroticism	**−9.64** *** (**−12.36 to −6.92**)		**−6.71** ** (**−11.02 to −2.41**)	**0.77** *** (**0.58–0.95**)		**0.66** *** (**0.37–0.96**)
Internalizing symptoms		**−9.14** *** (**−11.99 to −6.30**)	−3.84**** (−8.21 to 0.54)		**0.66** *** (**0.46–0.86**)	0.14 (−0.16 to 0.44)
Interactions						
Neur. × IP within	−0.11 (−0.25 to 0.03)		−0.00 (−0.23 to 0.22)	0.00 (−0.01 to 0.01)		−0.01 (−0.02 to 0.01)
Neur. × EP within	0.17 (−0.25 to 0.59)		0.40 (−0.30 to 1.09)	−0.01 (−0.04 to 0.02)		−0.03 (−0.07 to 0.02)
Int. × IP within		−0.12**** (−0.25 to 0.01)	−0.13 (−0.34 to 0.09)		0.01 (−0.00 to 0.01)	0.01 (−0.00 to 0.03)
Int. × EP within		0.02 (−0.39 to 0.43)	−0.27 (−0.94 to 0.39)		0.00 (−0.03 to 0.03)	0.02 (−0.03 to 0.07)

*Note:* Standard errors appear in parentheses. Probability values reported from Type III ANOVAs using Satterthwaite's method for estimating degrees of freedom. Negative affect was square root transformed to adjust for skew.

Abbreviations: EP = external‐present; IP = internal‐past; TC = thought content.

** *p* < 0.01.

*** *p* < 0.001.

**** *p* < 0.10.

#### 3.3.3 Direct Comparison of Neuroticism Versus Internalizing

To test whether neuroticism or internalizing symptoms had unique effects in predicting daily negative or positive affect, Model 5 included both neuroticism and internalizing, as well as their respective interactions with thought content. Evidence from model comparisons and fixed effects estimates showed that neuroticism, but not internalizing symptoms, had a unique effect on daily affect. As shown in Table 3, the combined model (Model 5) improved model fit over the internalizing model, but not the neuroticism model. For both positive and negative affect, all effects observed in Models 3 and 4 remained significant in Model 5 with similar point estimates, except for internalizing symptoms, which were no longer significant in the positive affect model, *γ* = −3.84 [−8.21, 0.54], *p* = 0.085, and the negative affect model, *γ* = 0.14 [−0.16, 0.44], *p* = 0.368. In other words, after accounting for severity of internalizing symptoms, neuroticism uniquely predicted higher levels of negative affect and lower levels of positive affect in daily life, but the reverse did not hold. No cross‐level interactions were significant in the combined model (Model 5) for positive or negative affect.

#### 3.3.4 Neuroticism Facets

A secondary model (Model 6) was calculated to test shared and unique effects of the facets of self‐consciousness and angry hostility, which were previously identified as being relevant in the internalizing spectrum and independently associated with interpersonal functioning (Fournier et al. 2019). Because of the increase in model parameters from Model 5 to 6 and because of the secondary nature of this model, we only examined main effects of self‐consciousness and angry hostility (interaction effects for all 6 neuroticism facets are probed in exploratory analyses below). As shown in Table 5, self‐consciousness uniquely predicted lower positive affect, while angry‐hostility uniquely predicted higher negative affect. This effect for angry hostility should be interpreted with caution due to the lower replicability of *p*‐values near 0.05 (Simonsohn et al. 2014) and the inclusion of multiple correlated predictors in this model. The effects of internalizing symptoms were significant in these models, *p* ≤ 0.008, indicating that self‐consciousness and angry hostility do not fully account for the internalizing symptoms' effect on daily emotional experience in the same way that the broader neuroticism dimension did in Model 5 above.

**TABLE 5:** Neuroticism facets and internalizing psychopathology predicting daily positive and negative affect: fixed effects estimates with 95% confidence intervals.

	Daily positive affect	Daily negative affect
Level 1 (within‐person)		
Internal‐past TC	**−0.20** ** (**−0.33 to −0.046**)	**0.05** *** (**0.04–0.06**)
External‐present TC	**0.77** *** (**0.38–1.17**)	−0.01 (−0.04 to 0.02)
Level 2 (between‐person)		
Internal‐past TC	−0.12 (−0.44 to 0.20)	**0.06** *** (**0.04–0.08**)
External‐present TC	**1.02** ** (**0.41–1.63**)	0.03 (−0.02 to 0.07)
Self‐consciousness	**−5.81** ** (**−9.60 to −2.02**)	0.21 (−0.07 to 0.48)
Angry‐hostility	−0.09 (−3.39 to 3.22)	**0.26** * (**0.02–0.50**)
Internalizing symptoms	**−5.48** ** (**−9.21 to −1.74**)	**0.37** ** (**0.10–0.64**)

*Note:* Standard errors appear in parentheses. Probability values reported from Type III ANOVAs using Satterthwaite's method for estimating degrees of freedom. Bold indicates significant tests at *p* <.05.

Abbreviations: EP = external‐present; IP = internal‐past; TC = thought content.

* *p* < 0.05.

** *p* < 0.01.

*** *p* < 0.001.

Exploratory analyses examined the effects of all six neuroticism facets, in models paralleling Model 5 with a neuroticism facet substituted for the overall neuroticism score (i.e., each model included effects for internalizing symptoms and a single neuroticism facet). Results are shown in Supporting Information S5. Because these analyses were exploratory and not part of a nested model sequence, the significance level was set at *α* = 0.008 (0.05/6 facet models). The facets of self‐consciousness and vulnerability predicted lower positive affect (Table S5.1), while the facets of anxiety, depression, angry hostility, and vulnerability predicted higher negative affect (Table S5.2). No facet × thought content interactions were significant.

#### 3.3.5 Sensitivity Analyses

Two sets of sensitivity analyses were conducted (see “Data Analysis” section above). When influential outliers were omitted (Tables S3.1 and S3.2), effects were similar except that the effect of internalizing was somewhat stronger and remained significant in the combined Model 5 predicting positive affect, *γ* = −6.61 [−11.02, −2.20], *p* = 0.003. When participants with extreme TC values were omitted (Tables S3.3 and S3.4), cross‐level interactions were larger than in main models. As a result, the internalizing symptoms × internal‐past TC interaction was a barely significant predictor of both positive affect, *γ* = −0.13 [−0.26, −0.00], *p* = 0.046, and negative affect, *γ* = 0.01 [0.00, 0.02], *p* = 0.049.

## 4 Discussion

This study explored relationships between thought content and affect in daily life, and the potential roles of neuroticism and internalizing symptoms in moderating those relationships. Two dimensions of thought content in daily life could be distinguished at both the between‐ and within‐person levels. On days when participants thought more about problems, emotions, and the past (internal‐past), they experienced more negative affect and less positive affect. By contrast, on days when participants thought more about others, external events, and the present (external‐present), they experienced more positive affect. These processes were largely independent of neuroticism and internalizing symptoms, which predicted higher negative affect and lower positive affect irrespective of daily thought content. This study has important implications for modeling thought content and affect in daily life, understanding the shared and unique effects of neuroticism and internalizing symptoms, and suggesting potential mechanisms of change.

### 4.1 Thought Content and Affect in Daily Life

Exploratory and multilevel confirmatory factor analysis found that thought content in daily life separated into two dimensions capturing thoughts about internal experience and the past versus thoughts about external experience and the present. Interestingly, each dimension collapses internal versus external orientation with past versus future focus. In an initial exploratory factor analysis (see Table 2), thoughts about the future appeared to load together with thoughts about internal experience and the past. This is consistent with models of repetitive negative thinking that combine worry (future focused) and rumination (past focused) as one transdiagnostic construct (McEvoy et al. 2013). However, when the initial exploratory model was respecified as a multilevel confirmatory factor model, thoughts about the future no longer cohered with thoughts about problems, emotions, and the past. MCFA loadings and model modification indices suggested that thoughts about the future corresponded with internal‐past thinking at the between‐person level, but not at the within‐person level. Thus, although worry and rumination may tend to bother the same people, they may bother those people in different ways on different days. Further research with a larger pool of thought content items would be valuable for identifying other kinds of thought content that may co‐vary with future‐oriented thinking in daily life.

Overall, internal‐past and external‐future thoughts were more distinct at the within‐person level than at the between‐person level (factor covariances −0.05 and 0.61, respectively). In other words, participants who tended to report higher levels of internal‐past thought content also tended to report higher levels of external‐present thought content. This could reflect an overall need for cognition (Cacioppo and Petty 1982), which would increase dispositional tendencies toward all types of thought content. At the between‐person level, internal‐past and external‐present thoughts both had bivariate relationships with negative affect (Table 2). This would be consistent with the proposal that excessive self‐generated thought (“thinking too much”) is a driving force behind neuroticism and depression (Perkins et al. 2015). Notably, however, when both types of thought content were included in the same models (Table 4), only internal‐past thoughts predicted negative affect. This provides further evidence that self‐generated thoughts are not all alike (see, e.g., Hoffmann et al. 2016): a tendency to think about internal‐past topics accompanies negative affectivity, over and above the shared effects of excessive self‐generated thought.

At the within‐person level, daily variation in external‐present thinking was unrelated to daily variation in internal‐past thinking. Daily external‐present thoughts also co‐occurred with higher positive affect, while daily internal‐past thoughts co‐occurred with lower positive affect and higher negative affect (Figure 2). Fluctuations in these two types of thought content seemed to play distinct roles in daily affective experience. Fluctuation in internal‐past thinking may reflect rumination, which has been shown to increase negative affect and decrease positive affect in daily life even in nonclinical samples (Brans et al. 2013). By contrast, fluctuation in external‐present thinking may reflect mindful awareness of the present moment, which has been shown to sustain positive affect in daily life (Rowland et al. 2020). Potential clinical implications for these findings will be discussed below.

### 4.2 Unique Role of Neuroticism and Its Facets

Neuroticism and internalizing psychopathology had somewhat asymmetrical roles in everyday cognitive‐affective experience. The effect of internalizing psychopathology on daily affect was fully accounted for by neuroticism. By contrast, neuroticism had unique effects on positive and negative affect in daily life, over and above the effect of internalizing symptoms. Internalizing symptoms may be acute manifestations of some aspects of underlying trait neuroticism. However, neuroticism had a broader effect on daily emotional experience, extending beyond internalizing symptoms alone. Examining the facet‐level analyses, this is likely because neuroticism's predictive power partly derives from facets that closely resemble internalizing symptoms (e.g., depression, anxiety), but partly from facets that do not (e.g., self‐consciousness, angry hostility, vulnerability). This pattern is consistent with the theoretical conceptualization of neuroticism as a general tendency to experience aversive emotional states, in contrast to internalizing symptoms, which are typically thought of as episodic fear‐ and distress‐related phenomena that wax and wane over time (DeYoung et al. 2022). It is also consistent with findings that psychotherapy and medications change neuroticism over and above their effect on internalizing symptoms (Fournier et al. 2020, 2019; Sauer‐Zavala et al. 2021; Tang et al. 2009). The study of affective dynamics in daily life appears to be a viable way forward in understanding differential effects of neuroticism and internalizing psychopathology. We hope that the current manuscript might (1) assist researchers in parsing shared and nonshared variance between internalizing symptoms and neuroticism; and (2) stimulate more research probing non‐overlapping aspects of neuroticism and internalizing within persons.

Moreover, specific facets of neuroticism were related to specific kinds of aversive affect in theoretically coherent ways. Self‐consciousness was associated with lower positive affect, perhaps because self‐conscious emotions lead to inhibition of behaviors that would otherwise spark positive affective experiences (e.g., spending time with friends, engaging in meaningful work or enjoyable hobbies). By contrast, angry hostility was associated with higher negative affect, perhaps because expressions of hostility or aggression provoke interpersonal friction, conflict, or rejection. These findings—particularly for angry hostility—are more prone to Type I error due to the number of correlated model terms in these models, and results should be considered speculative until replicated in independent samples. Nevertheless, neuroticism facets seemed to provide important additional explanatory power for understanding daily affective experience. In exploratory analyses, the facet of vulnerability was noteworthy, predicting both higher negative affect and lower positive affect. This facet may be valuable to examine in future confirmatory work relating to emotion and cognition in daily life.

Contrary to our hypotheses, we did not observe interactions between neuroticism, internalizing symptoms, and thought content in predicting positive or negative affect. The lack of significant interactions could be a Type II error, as true interaction effect sizes tend to be much smaller than true main effect sizes (Enders et al. 2025; Hyatt et al. 2022; Vize et al. 2023). One interaction term, internalizing × internal‐past TC predicting positive affect, neared significance in the primary analysis (Table 4, Positive Affect Model 4; *p* = 0.063) and reached significance in a sensitivity analysis. This suggests a potential lead for future research, but the present study provides no direct evidence for interactions. Unmeasured dimensions of variation in daily thought content (e.g., future‐oriented thinking; positivity vs. negativity) or higher‐frequency processes (e.g., measured in minutes or hours rather than days) could be important factors to consider. Methodologically, a larger Level 1 sample size (more observations per person) would have increased this study's ability to detect cross‐level interactions, and future work on this subject would benefit from larger Level 1 sample sizes. Additionally, more frequent sampling might uncover interactions operating on timescales of hours rather than days, while limiting any biases in retrospective recall.

Substantively, a lack of interaction would suggest that neuroticism and internalizing symptoms exert relatively consistent effects in daily affective experience regardless of what one thinks about during the day. This would imply that within‐person differences between neuroticism and internalizing (e.g., differences in treatment effects) may operate through overall affective experience rather than specific affective reactions to thought content.

### 4.3 Potential Clinical Implications

The present study's results suggest some speculation about possible clinical applications. If self‐consciousness is more related to deficient positive affect in daily life, and angry hostility is more related to excessive negative affect in daily life, this suggests a possible avenue toward personalizing treatment based on a particular individual's neuroticism facet profile. Plausibly, individuals higher in self‐consciousness might benefit from behavioral strategies to increase positive affect (e.g., behavioral activation); whereas individuals high in angry hostility might benefit from cognitive strategies to decrease negative affect (e.g., cognitive restructuring; Mausbach et al. 2009).

Research has also shown that mindfulness protects against depression in individuals high in neuroticism (Barnhofer et al. 2011). Although mindfulness was not assessed in the present study, the thought content factors identified in the present study may have implications for mindfulness‐based interventions (e.g., mindfulness‐based cognitive therapy, acceptance and commitment therapy, acceptance‐based behavioral therapy). These interventions cultivate greater present‐moment awareness and support mindfully disengaging from worry and rumination. The mechanisms identified in the present study seem to map onto the proposed mechanisms of change in mindfulness‐based therapies. We might say that these interventions promote disengagement from internal‐past thought content and engagement with external‐present thought content to reduce negative affect and increase positive affect. The ability to quantify these processes in daily life could be beneficial in future research on mechanisms of change in mindfulness‐based interventions.

### 4.4 Study Limitations

The present study's small within‐person sample size may have limited its ability to detect relevant cross‐level interaction effects, particularly given that: (1) interaction effect sizes tend to be quite small; (2) observed effect sizes for relationships between thought content and affect (Table 3) were larger at the between‐person level than the within‐person level; and (3) neuroticism and internalizing symptoms were strongly correlated. While this study failed to detect effects, point estimates were in the direction of the hypothesized interaction effects, suggesting that these effects could potentially be detectable with a larger sample size (particularly with more observations per person). Additionally, more frequent assessment of affect would allow more detailed analysis of affective dynamics within each day, such as affective variability and reactivity (Mukherjee et al. 2023) or affective inertia and propensity to switch from positive to negative affect (Rowland et al. 2020). Finally, the sample included a dimensional representation of internalizing severity but did not consider categorical diagnoses (e.g., potential differences between fear‐based vs. distress‐based internalizing) (Watson et al. 2022). Relevant limitations to generalizability included recruitment from one geographic area (Midwest) in the United States (which constrains generalization to other populations); exclusion of bipolar, psychotic, and substance use disorders; and restriction to a single developmental stage (early adulthood).

## Author Contributions

Conceptualization: H.R.C. and J.C.F.; Methodology: H.R.C., A.G.C.W., and J.C.F.; Formal analysis: H.R.C.; Investigation: J.C.F., S.L.P., and D.M.; Writing – original draft: H.R.C.; Writing – review and editing: All authors; Visualization: H.R.C.; Supervision: J.C.F.; Funding acquisition: J.C.F.

## Conflicts of Interest

J.C.F. has received royalties from Guilford Press. No other authors have conflicts of interest to disclose.

## Supporting information


**Data S1:** jopy70026‐sup‐0001‐DataS1.docx.

## Data Availability

The deidentified data and analysis code supporting this study are publicly available at https://osf.io/5vy6m/. Study materials are either freely available online or subject to copyright (NEO‐PI‐R). The novel Daily Thought Content Questionnaire described in this manuscript can be found at https://osf.io/5vy6m/. Supporting Information have been submitted with this manuscript and will appear on the journal's website. This study was not preregistered.
