# Supplementary material for: Neuroticism, Internalizing Psychopathology, and Affective Reactions to Thought Content in Daily Life
Source: J Pers. 2025 Oct 20;94(4):625–39. doi: 10.1111/jopy.70026 (PMC13359292; doi:10.1111/jopy.70026)
Supplement: Supplementary file 1 — Data S1: jopy70026‐sup‐0001‐DataS1.docx. [file JOPY-94-625-s001.docx]

**Supplemental Material for**

**Neuroticism, Internalizing Psychopathology,
and Affective Reactions to Thought Content in Daily Life**

Henry R. Cowan^1,2^, Aidan G. C. Wright^3^, Sarah L. Pedersen^4^, Dahlia Mukherjee^5^**,** Sophie Lazarus^2^, Jay C. Fournier^2^

^1^Psychology, Michigan State University

^2^Psychiatry and Behavioral Health, The Ohio State University

^3^Psychology, University of Michigan

^4^Psychiatry, University of Pittsburgh

^5^Psychiatry and Behavioral Health, Penn State College of Medicine, Pennsylvania State University

Correspondence: Henry R. Cowan, hrcowan@msu.edu

**S1. Multilevel Power Analysis**

Power analyses were conducted for primary analyses in the main text (Models 3-5) in the {mlmpower} *R* package. Analyses examined a range of plausible effect sizes for within-person effects, between-person effects, and cross-level effects. ICC was set to .65 in all models to approximate the observed ICCs in the dataset. Random slope effect size was set to .03 following estimates given by Enders et al (2023). The between-person components of thought content were weighted at 50% the weight of other predictors, to reflect the narrower nature of these constructs, i.e., they are between-person only variance components that together make up two indicators of a single domain (daily thought content). Note that simulated power analyses provide rough benchmarks rather than exact tests of achieved power due to the number of assumptions made about the data structure. Power analyses were calculated via Monte Carlo simulation with 500 repetitions per data structure, with Level 2 *n*=119 and within-cluster Level 1 *n*=7.

Effect sizes in these analyses are the overall effect for all independent variables at a given level, e.g., between-person effect size *R*^2^ = .13 indicates that all between-person variables collectively account for 13% of variance in the outcome variable. Target effect sizes (indicated in bold in Table S1.1) were *R*^2^ =.13 at within and between person levels, cross-level *R*^2^ =.013 for Models 3 & 4, and cross-level *R*^2^ =.026 for Model 5. A larger total cross-level effect size is expected in Model 5 model because it includes 4 cross-level interactions rather than 2 in Models 3 & 4. Note that Enders et al (2023) give a range of .01 to .05 for this cross-level effect size. Table S1.1 shows target effect sizes in bold alongside a range of other plausible effect sizes.

| ***Table S1.1***  Power in Models 3-5 for various combinations of within, between, and cross-level effect sizes | | | | | | |
| --- | --- | --- | --- | --- | --- | --- |
| **Effect sizes (*R*^2^)** | | | **Power, Model 3 & 4** | | **Power, Model 5** | |
| **Within** | **Between** | **Product** | **Main effects** | **Interactions** | **Main effects** | **Interactions** |
| 0.065 | 0.065 | 0.013 | ≥.63 | ≥.89 | ≥.40 | ≥.48 |
| 0.13 | 0.065 | 0.013 | ≥.64 | ≥.92 | ≥.38 | ≥.56 |
| 0.26 | 0.065 | 0.013 | ≥.63 | ≥.99 | ≥.37 | ≥.83 |
| 0.065 | 0.13 | 0.013 | ≥.92 | ≥.88 | ≥.67 | ≥.48 |
| 0.13 | 0.13 | 0.013 | **≥.94** | **≥.96** | ≥.59 | ≥.58 |
| 0.26 | 0.13 | 0.013 | ≥.92 | ≥.99 | ≥.68 | ≥.83 |
| 0.065 | 0.26 | 0.013 | ≥.99 | ≥.89 | ≥.89 | ≥.47 |
| 0.13 | 0.26 | 0.013 | ≥.99 | ≥.90 | ≥.97 | ≥.58 |
| 0.26 | 0.26 | 0.013 | ≥.99 | ≥.99 | ≥.96 | ≥.82 |
| 0.065 | 0.065 | 0.026 | ≥.64 | ≥.99 | ≥.39 | ≥.76 |
| 0.13 | 0.065 | 0.026 | ≥.92 | ≥.99 | ≥.38 | ≥.86 |
| 0.26 | 0.065 | 0.026 | ≥.60 | ≥.99 | ≥.36 | ≥.99 |
| 0.065 | 0.13 | 0.026 | ≥.92 | ≥.99 | ≥.69 | ≥.81 |
| 0.13 | 0.13 | 0.026 | ≥.92 | ≥.99 | **≥.68** | **≥.88** |
| 0.26 | 0.13 | 0.026 | ≥.93 | ≥.99 | ≥.66 | ≥.99 |
| 0.065 | 0.26 | 0.026 | ≥.99 | ≥.99 | ≥.96 | ≥.80 |
| 0.13 | 0.26 | 0.026 | ≥.99 | ≥.99 | ≥.97 | ≥.88 |
| 0.26 | 0.26 | 0.026 | ≥.99 | ≥.99 | ≥.86 | ≥.99 |

**S2. Measure Development and Multilevel Confirmatory Factor Analysis of Thought Content Items**

The daily thought content measure was developed for this study, due to there being no available measure of thought content in daily life. Items were generated collaboratively by the research team and decisions about item content were made by consensus. The broader study was focused on personality traits, internalizing symptoms, and cognitive processing of self- and other-related stimuli. Initial constructs of interest were repetitive negative thinking (rumination and worry) and self- and other-focused thought, which are known to be relevant for neuroticism and internalizing psychopathology. To assess these constructs briefly in a daily diary survey, four items were initially generated for thoughts about the self, others, the past, and the future. Through consensus discussion, the research team felt that the self and other categories were too broad, so these items were split into broad domains of internal (feelings/emotions, problems/challenges) and external experience (close others, acquaintances, and external events). Additionally, a present item was added to provide an explicit contrast to the future and past items. This final list of eight items (see main text, Table 3) was administered to participants. Scale structure and internal consistency was assessed via EFA and multilevel CFA (see main text and Table S2.1 below), with two items being dropped to produce a final scale with six items. The length limitations in the daily diary survey limited the measures that could be administered to assess convergent and discriminant validity. Within-person correlations with daily positive and negative affect (main text Table 2) indicate that the daily thought content scales have theoretically-consistent relationships with positive and negative affect (see main text, p. 18-19).

| **Table S2.1**  Multilevel confirmatory factor analysis of daily thought content items | | | | | | | |  |
| --- | --- | --- | --- | --- | --- | --- | --- | --- |
| Factors Within | Factors Between | Items | Notes | CFI | RMSEA | SRMR Within | SRMR Between | |
| 1 | 1 | 8 | - | .682 | .073 | **.075** | .093 | |
| 2 | 1 | 8 | - | .759 | .064 | **.064** | .092 | |
| 1 | 2 | 8 | - | .723 | .069 | **.075** | **.072** | |
| 2 | 2 | 8 | - | .784 | .061 | **.064** | **.070** | |
| 2 | 2 | 8 | “Close others”  allowed to cross-load | .813 | **.059** | **.056** | **.070** | |
| 2 | 2 | 8 | “Future”  allowed to cross-load | .814 | **.059** | **.058** | **.067** | |
| 2 | 2 | 8 | “Close others” and “future” allowed to cross-load | DNC | DNC | DNC | DNC | |
| 2 | 2 | 7 | “Future” dropped | .878 | **.048** | **.050** | **.063** | |
| 2 | 2 | 7 | “Close others” dropped | .808 | .063 | **.057** | **.068** | |
| 2 | 2 | 6 | “Close others” and “future” dropped | **.926** | **.041** | **.031** | **.067** | |
| DNC = “did not converge”. Results which met prespecified absolute fit criteria indicated in bold (CFI≥.90, RMSEA≤.06, SRMR≤.08). | | | | | | | |  |

**S3. Sensitivity Analyses**

Tables in this section correspond to Tables 4 and 5 in the main text. This first set of tables (Tables S2 and S3) show analyses omitting four participants identified as influential outliers, based on extreme values in residuals and influence (Cook’s Distance). As shown in Figures S1 and S2, four participants (3001, 3011, 3178, 3085) had residuals >2 SD from the mean (indicated by dashed lines) and large relative values on Cook’s distance. Models omitting these four participants are presented in Tables S2 and S3 below

**Figure S3.1**. Residuals and Cook’s distance in positive affect combined model (Model 5 in main text)


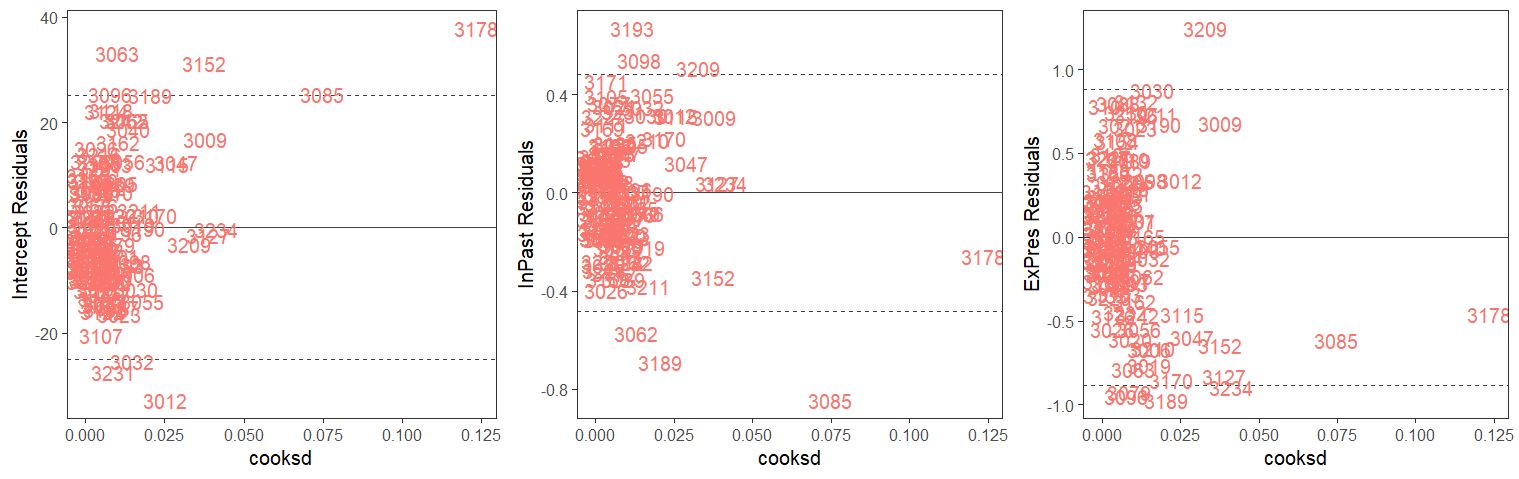


**Figure S3.2**. Residuals and Cook’s distance in negative affect combined model (Model 5 in main text)


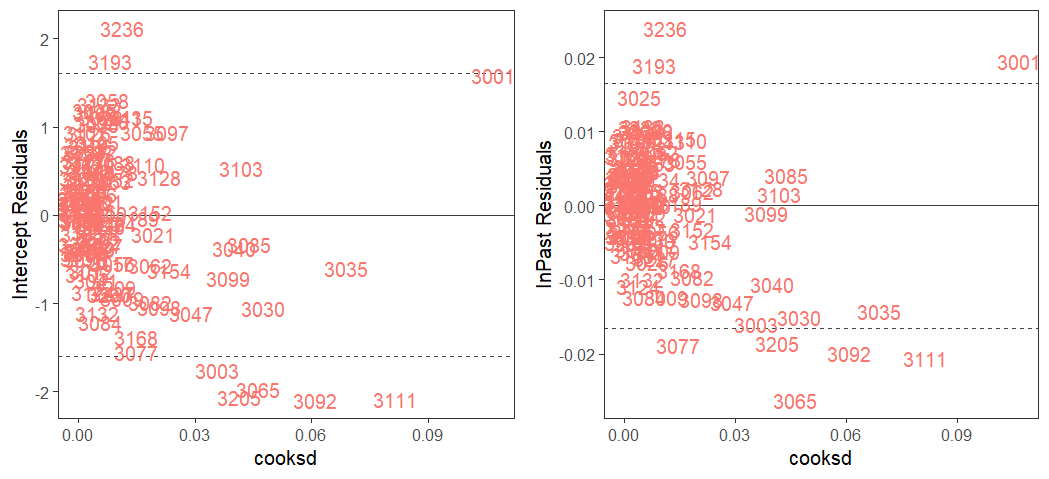


| ***Table S3.1. INFLUENTIAL OUTLIERS REMOVED***  Neuroticism, Internalizing, and Daily Thought Content Predicting Daily Positive and Negative Affect: Fixed Effects Estimates with 95% Confidence Intervals | | | | | | | |
| --- | --- | --- | --- | --- | --- | --- | --- |
|  | Daily Positive Affect | | |  | Daily Negative Affect | | |
|  | Model 3 | Model 4 | Model 5 |  | Model 3 | Model 4 | Model 5 |
| Level 1 (Within-person) |  |  |  |  |  |  |  |
| Intercept | **36.83*** (34.23 – 39.44)** | **36.51*** (33.94 – 39.07)** | **36.66*** (34.13 – 39.18)** |  | **3.74*** (3.56 – 3.91)** | **3.76*** (3.57 – 3.95)** | **3.74*** (3.57 – 3.91)** |
| IP Thinking (within) | **-0.17* (-0.30 – -0.04)** | **-0.17** (-0.30 – -0.04)** | **-0.17** (-0.30 – -0.04)** |  | **0.05*** (0.04 – 0.06)** | **0.05*** (0.04 – 0.06)** | **0.05*** (0.04 – 0.06)** |
| EP Thinking (within) | **0.76*** (0.35 – 1.16)** | **0.78*** (0.37 – 1.18)** | **0.74*** (0.33 – 1.15)** |  | -0.01 (-0.04 – 0.02) | -0.01 (-0.04 – 0.02) | -0.01 (-0.03 – 0.02) |
| Level 2 (Between-person) |  |  |  |  |  |  |  |
| IP Thinking (between) | -0.18 (-0.54 – 0.17) | -0.17 (-0.52 – 0.18) | -0.13  (-0.47 – 0.21) |  | **0.05*** (0.02 – 0.07)** | **0.05*** (0.03 – 0.08)** | **0.05*** (0.02 – 0.07)** |
| EP Thinking (between) | **1.20*** (0.53 – 1.86)** | **1.28*** (0.64 – 1.93)** | **1.22*** (0.58 – 1.86)** |  | **0.06* (0.01 – 0.10)** | 0.05 (-0.00 – 0.09) | **0.05* (0.01 – 0.10)** |
| Neuroticism | **-9.76*** (-12.59 – -6.93)** |  | **-4.78* (-9.10 – -0.45)** |  | **0.85*** (0.67 – 1.04)** |  | **0.72*** (0.42 – 1.02)** |
| Internalizing |  | **-10.37*** (-13.21 – -7.52)** | **-6.61** (-11.02 – -2.20)** |  |  | **0.75*** (0.54 – 0.96)** | 0.18 (-0.13 – 0.48) |
| Interactions |  |  |  |  |  |  |  |
| Neur. x Daily IP | -0.08 (-0.22 – 0.06) |  | 0.00 (-0.22 – 0.23) |  | 0.00 (-0.01 – 0.01) |  | -0.00 (-0.02 – 0.01) |
| Neur. x Daily EP | 0.20 (-0.23 – 0.63) |  | 0.39 (-0.32 – 1.10) |  | -0.01 (-0.04 – 0.02) |  | -0.02 (-0.07 – 0.03) |
| Int. x Daily IP |  | -0.08 (-0.21 – 0.05) | -0.09 (-0.31 – 0.12) |  |  | 0.00 (-0.00 – 0.01) | 0.01 (-0.01 – 0.02) |
| Int. x Daily EP |  | 0.07 (-0.34 – 0.49) | -0.22 (-0.91 – 0.46) |  |  | -0.00 (-0.03 – 0.03) | 0.02 (-0.03 – 0.06) |
| *Note*: IP = Internal-past; EP = External-Present. Standard errors appear in parentheses. Probability values reported from Type III ANOVAs using Satterthwaite’s method for estimating degrees of freedom. Negative affect was square root transformed to adjust for skew. ^†^*p* <.10, **p* < .05, ***p* < .01, ****p* < .001 | | | | | | | |
|  | | | | | | | |

| ***Table S3.2. INFLUENTIAL OUTLIERS REMOVED***  Neuroticism Facets and Internalizing Psychopathology Predicting Daily Positive and Negative Affect: Fixed Effects Estimates | | | |
| --- | --- | --- | --- |
|  |  | Daily Positive Affect | Daily Negative Affect |
| Level 1 (Within-person) |  |  |  |
| Intercept |  | **36.62*** (34.11 – 39.14)** | **3.76*** (3.57 – 3.94)** |
| IP Thinking (within) |  | **-0.17** (-0.29 – -0.04)** | **0.05*** (0.04 – 0.06)** |
| EP Thinking (within) |  | **0.73*** (0.33 – 1.14)** | -0.01 (-0.04 – 0.02) |
| Level 2 (Between-person) |  |  |  |
| IP Thinking (between) |  | -0.15 (-0.49 – 0.20) | **0.05*** (0.02 – 0.07)** |
| EP Thinking (between) |  | **1.21*** (0.56 – 1.85)** | **0.05* (0.01 – 0.10)** |
| Self-consciousness |  | **-4.68* (-8.49 – -0.86)** | **0.30* (0.02 – 0.57)** |
| Angry-hostility |  | 0.41 (-2.85 – 3.66) | 0.21^†^ (-0.02 – 0.45) |
| Internalizing |  | **-7.36*** (-11.14 – -3.57)** | **0.44** (0.16 – 0.71)** |
| Interactions |  |  |  |
| Self-Cons. x Daily IP |  | 0.02 (-0.17 – 0.21) | 0.00 (-0.01 – 0.01) |
| Self-Cons. x Daily EP |  | 0.53 (-0.10 – 1.15) | 0.00 (-0.04 – 0.05) |
| Ang. Host. x Daily IP |  | -0.10 (-0.26 – 0.07) | -0.00 (-0.01 – 0.01) |
| Ang. Host. x Daily EP |  | 0.01 (-0.51 – 0.52) | -0.01 (-0.05 – 0.02) |
| Internalizing x Daily IP |  | -0.05 (-0.23 – 0.12) | 0.00 (-0.01 – 0.02) |
| Internalizing x Daily EP |  | -0.27 (-0.86 – 0.31) | 0.00 (-0.04 – 0.05) |
| *Note*: IP = Internal-past; EP = External-Present. Standard errors appear in parentheses. Probability values reported from Type III ANOVAs using Satterthwaite’s method for estimating degrees of freedom. **p* < .05, ***p* < .01, ****p* < .001 | | | |

The following models in Tables S4 and S5 omit three participants who had extremely high scores on one or more of the thought content factors, as highlighted in Figures S3 and S4 below.

**Figure S3.3**. Between-person external-present thought content scores, with extreme values highlighted.


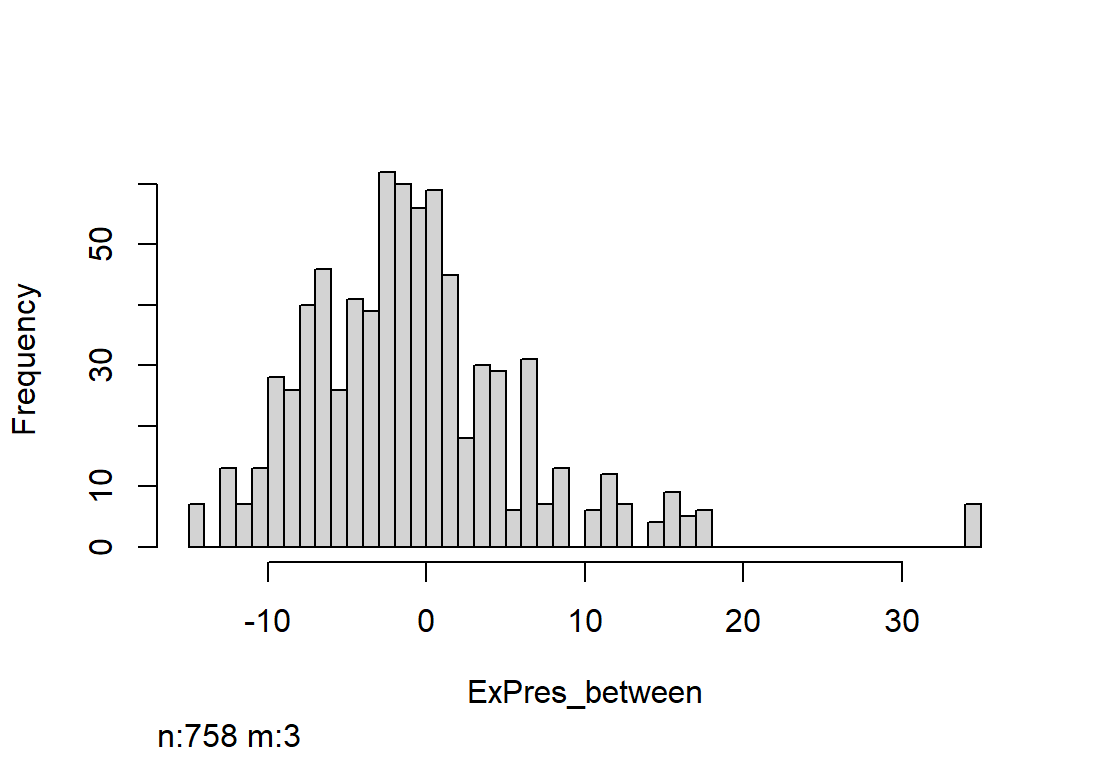


**Figure S3.4**. Between-person internal-past thought content scores, with extreme values highlighted.


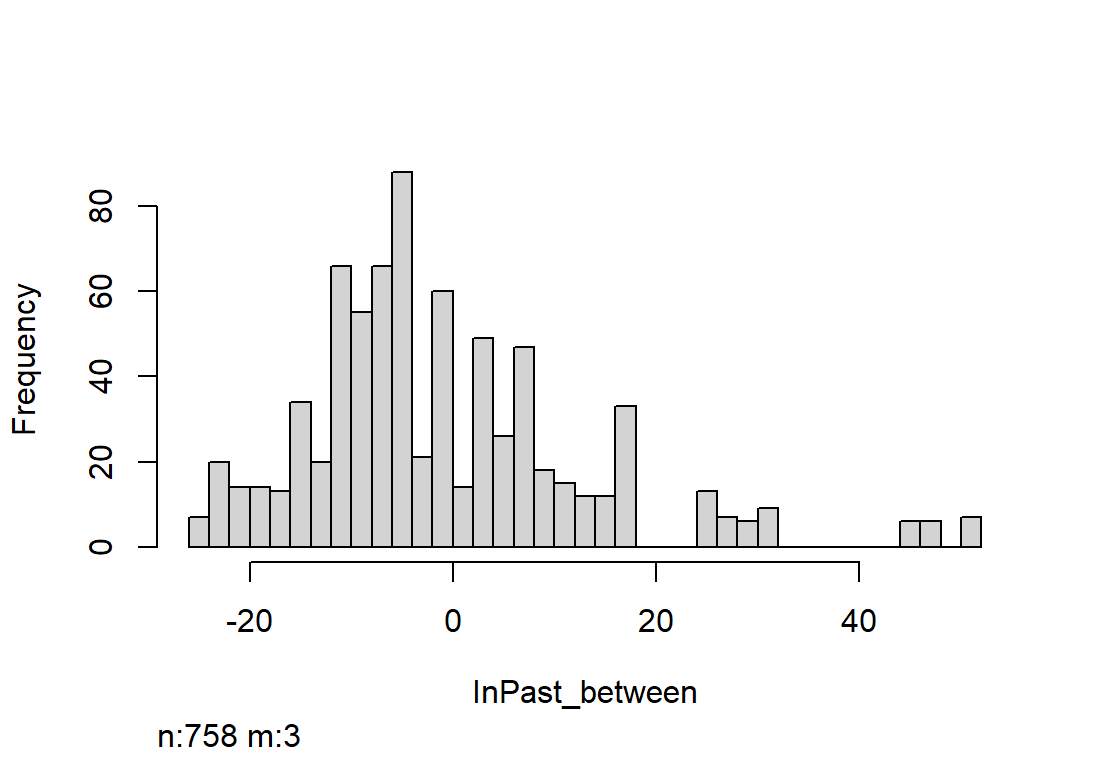


| ***Table S3.3. EXTREME VALUES REMOVED***  Neuroticism, Internalizing, and Daily Thought Content Predicting Daily Positive and Negative Affect: Fixed Effects Estimates with 95% Confidence Intervals | | | | | | | |
| --- | --- | --- | --- | --- | --- | --- | --- |
|  | Daily Positive Affect | | |  | Daily Negative Affect | | |
|  | Model 3 | Model 4 | Model 5 |  | Model 3 | Model 4 | Model 5 |
| Level 1 (Within-person) |  |  |  |  |  |  |  |
| Intercept | **37.53*** (34.83 – 40.22)** | **37.27*** (34.50 – 40.05)** | **37.48*** (34.80 – 40.16)** |  | **3.73*** (3.55 – 3.91)** | **3.75*** (3.55 – 3.95)** | **3.73*** (3.55 – 3.91)** |
| IP Thinking (within) | **-0.18** (-0.31 – -0.05)** | **-0.18** (-0.32 – -0.05)** | **-0.18** (-0.32 – -0.05)** |  | **0.05*** (0.04 – 0.06)** | **0.05*** (0.04 – 0.06)** | **0.05*** (0.04 – 0.06)** |
| EP Thinking (within) | **0.70** (0.30 – 1.09)** | **0.71*** (0.32 – 1.11)** | **0.67** (0.27 – 1.07)** |  | -0.00 (-0.03 – 0.02) | -0.01 (-0.03 – 0.02) | -0.00 (-0.03 – 0.03) |
| Level 2 (Between-person) |  |  |  |  |  |  |  |
| IP Thinking (between) | -0.05 (-0.41 – 0.31) | -0.07 (-0.44 – 0.30) | -0.02 (-0.38 – 0.34) |  | **0.06*** (0.03 – 0.08)** | **0.06*** (0.04 – 0.09)** | **0.06*** (0.03 – 0.08)** |
| EP Thinking (between) | **1.00** (0.33 – 1.68)** | **0.95** (0.26 – 1.63)** | **0.97** (0.31 – 1.64)** |  | 0.03 (-0.01 – 0.08) | 0.03 (-0.02 – 0.08) | 0.03 (-0.01 – 0.08) |
| Neuroticism | **-9.89*** (-12.80 – -6.98)** |  | **-6.93** (-11.47 – -2.39)** |  | **0.84*** (0.64 – 1.03)** |  | **0.72*** (0.41 – 1.02)** |
| Internalizing |  | **-9.00*** (-11.96 – -6.05)** | -3.77^†^ (-8.25 – 0.70) |  |  | **0.70*** (0.49 – 0.91)** | 0.15 (-0.15 – 0.45) |
| Interactions |  |  |  |  |  |  |  |
| Neur. x Daily IP | -0.13^†^ (-0.27 – 0.02) |  | -0.01 (-0.24 – 0.22) |  | 0.01 (-0.00 – 0.02) |  | -0.00 (-0.02 – 0.01) |
| Neur. x Daily EP | 0.15 (-0.27 – 0.57) |  | 0.53 (-0.17 – 1.22) |  | -0.00 (-0.03 – 0.03) |  | -0.03 (-0.08 – 0.02) |
| Int. x Daily IP |  | **-0.13* (-0.26 – -0.00)** | -0.14 (-0.35 – 0.08) |  |  | **0.01* (0.00 – 0.02)** | 0.01 (-0.00 – 0.03) |
| Int. x Daily EP |  | -0.06 (-0.46 – 0.35) | -0.44 (-1.11 – 0.22) |  |  | 0.01 (-0.02 – 0.04) | 0.03 (-0.02 – 0.07) |
| *Note*: IP = Internal-past; EP = External-Present. Standard errors appear in parentheses. Probability values reported from Type III ANOVAs using Satterthwaite’s method for estimating degrees of freedom. Negative affect was square root transformed to adjust for skew. ^†^*p* <.10, **p* < .05, ***p* < .01, ****p* < .001 | | | | | | | |

| ***Table S3.4. EXTREME VALUES REMOVED***  Neuroticism Facets and Internalizing Psychopathology Predicting Daily Positive and Negative Affect: Fixed Effects Estimates | | | |
| --- | --- | --- | --- |
|  |  | Daily Positive Affect | Daily Negative Affect |
| Level 1 (Within-person) |  |  |  |
| Intercept |  | **37.41*** (34.73 – 40.09)** | **3.74*** (3.55 – 3.93)** |
| IP Thinking (within) |  | **-0.18** (-0.31 – -0.05)** | **0.05*** (0.04 – 0.06)** |
| EP Thinking (within) |  | **0.66** (0.26 – 1.06)** | -0.00 (-0.03 – 0.02) |
| Level 2 (Between-person) |  |  |  |
| IP Thinking (between) |  | -0.07 (-0.44 – 0.29) | **0.06*** (0.03 – 0.08)** |
| EP Thinking (between) |  | **0.96** (0.29 – 1.63)** | 0.03 (-0.01 – 0.08) |
| Self-consciousness |  | **-6.11** (-10.04 – -2.17)** | 0.23 (-0.05 – 0.51) |
| Angry-hostility |  | 0.79 (-2.73 – 4.30) | **0.25* (0.01 – 0.50)** |
| Internalizing |  | **-5.30** (-9.20 – -1.41)** | **0.42** (0.14 – 0.69)** |
| Interactions |  |  |  |
| Self-Cons. x Daily IP |  | 0.04 (-0.15 – 0.23) | -0.00 (-0.01 – 0.01) |
| Self-Cons. x Daily EP |  | 0.57^†^ (-0.03 – 1.17) | -0.01 (-0.05 – 0.04) |
| Ang. Host. x Daily IP |  | -0.15^†^ (-0.32 – 0.02) | 0.00 (-0.01 – 0.01) |
| Ang. Host. x Daily EP |  | -0.00 (-0.51 – 0.51) | -0.00 (-0.04 – 0.04) |
| Internalizing x Daily IP |  | -0.09 (-0.27 – 0.08) | 0.01 (-0.00 – 0.02) |
| Internalizing x Daily EP |  | -0.42 (-0.99 – 0.16) | 0.01 (-0.03 – 0.05) |
| *Note*: IP = Internal-past; EP = External-Present. Standard errors appear in parentheses. Probability values reported from Type III ANOVAs using Satterthwaite’s method for estimating degrees of freedom. **p* < .05, ***p* < .01, ****p* < .001 | | | |

**S4. Reasons for Study Noncompletion**

An additional 124 individuals consented to screening but did not complete the study. Reasons for study noncompletion were as follows:

| ***Table S4***  Reasons for Study Noncompletion |  |
| --- | --- |
| **Noncompletion reason** | **N** |
| English language proficiency/IQ | 5 |
| Symptoms outside eligibility cut-offs | 9 |
| Not Interested/Scheduling Challenges | 9 |
| fMRI exclusions | 11 |
| COVID-19 Disruptions | 26 |
| Could not determine eligibility (e.g., participant did not complete required measure) | 31 |
| Current treatment/Substance use/Excluded mental health diagnosis | 33 |

**S5. Exploratory Models with all Six Neuroticism Facets**

These exploratory models parallel Model 5 in the main text, testing the effect of each neuroticism facet in the same model as internalizing symptoms. Because these analyses are exploratory and not part of a nested model sequence, the significance level for interpreting these models has been set at α = .008 (.05 / 6 facet-level models = .008), indicated in bold in the tables below.

| **Table S5.1 Neuroticism Facets, Internalizing Symptoms, and Thought Content Predicting *Daily Positive Affect*** | | | | | | | | | | | | |
| --- | --- | --- | --- | --- | --- | --- | --- | --- | --- | --- | --- | --- |
|  | **N1 Anxiety** | | **N2 Angry Hostility** | | **N3 Depression** | | **N4 Self-consciousness** | | **N5 Impulsivity** | | **N6 Vulnerability** | |
| *Predictors* | *Estimates* | *p* | *Estimates* | *p* | *Estimates* | *p* | *Estimates* | *p* | *Estimates* | *p* | *Estimates* | *p* |
| **Level 1 (Within-person)** | | | | | | | | | | | | |
| Internal-past TC | -0.06 (-0.64 – 0.52) | 0.844 | -0.17 (-0.30 – -0.04) | 0.010 | -0.44 (-1.00 – 0.12) | 0.122 | **-0.18 (-0.31 – -0.05)** | **0.005** | -0.38 (-0.82 – 0.07) | 0.096 | -0.26 (-0.74 – 0.21) | 0.273 |
| External-present TC | 0.28 (-1.59 – 2.14) | 0.772 | **0.75 (0.35 – 1.15)** | **<0.001** | 0.09 (-1.57 – 1.75) | 0.917 | **0.74 (0.34 – 1.14)** | **<0.001** | 0.60 (-0.79 – 1.98) | 0.398 | 0.25 (-1.24 – 1.74) | 0.742 |
| **Level 2 (Between-person)** | | | | | | | | | | | | |
| Internal-past TC | -0.11 (-0.43 – 0.20) | 0.482 | -0.07 (-0.40 – 0.26) | 0.696 | -0.10 (-0.42 – 0.22) | 0.543 | -0.13 (-0.44 – 0.18) | 0.426 | -0.12 (-0.44 – 0.21) | 0.480 | -0.07 (-0.38 – 0.24) | 0.647 |
| External-present TC | **1.01 (0.40 – 1.62)** | **0.001** | **1.01 (0.38 – 1.64)** | **0.002** | **1.05 (0.44 – 1.67)** | **0.001** | **1.03 (0.43 – 1.63**) | **0.001** | **1.06 (0.44 – 1.69)** | **0.001** | **1.00 (0.40 – 1.60)** | **0.001** |
| Neuroticism facet | -0.62 (-1.17 – -0.06) | 0.029 | -1.40 (-4.67 – 1.88) | 0.403 | -0.66 (-1.22 – -0.09) | 0.023 | **-5.85 (-9.45 – -2.25)** | **0.001** | -0.40 (-0.92 – 0.11) | 0.122 | **-0.98 (-1.57 – -0.39)** | **0.001** |
| Internalizing symptoms | **-5.78 (-9.89 – -1.66)** | **0.006** | **-8.53 (-11.8 – -5.27)** | **<0.001** | -4.42 (-9.35 – 0.52) | 0.079 | **-5.00 (-8.74 – -1.27)** | **0.009** | **-7.98 (-11.2 – -4.80)** | **<0.001** | -4.88 (-8.63 – -1.14) | 0.011 |
| **Cross-level interactions** | | | | | | | | | | | | |
| Facet x IP within | -0.01 (-0.03 – 0.02) | 0.672 | -0.13 (-0.29 – 0.02) | 0.097 | 0.01 (-0.01 – 0.04) | 0.348 | -0.02 (-0.20 – 0.16) | 0.849 | 0.01 (-0.01 – 0.04) | 0.365 | 0.01 (-0.02 – 0.04) | 0.711 |
| Facet x EP within | 0.02 (-0.06 – 0.11) | 0.584 | 0.14 (-0.34 – 0.63) | 0.562 | 0.04 (-0.05 – 0.12) | 0.404 | 0.53 (-0.05 – 1.10) | 0.072 | 0.01 (-0.07 – 0.09) | 0.798 | 0.04 (-0.06 – 0.13) | 0.473 |
| Int. x IP within | -0.10 (-0.28 – 0.08) | 0.261 | -0.06 (-0.21 – 0.09) | 0.418 | -0.22 (-0.45 – 0.01) | 0.064 | -0.12 (-0.29 – 0.06) | 0.180 | -0.16 (-0.31 – -0.01) | 0.041 | -0.15 (-0.33 – 0.03) | 0.112 |
| Int x EP within | -0.10 (-0.68 – 0.48) | 0.739 | -0.04 (-0.51 – 0.43) | 0.868 | -0.23 (-0.94 – 0.49) | 0.536 | -0.32 (-0.88 – 0.24) | 0.260 | -0.01 (-0.49 – 0.46) | 0.953 | -0.13 (-0.71 – 0.45) | 0.665 |
| *Note*: Bold indicates *p* < .008 (.05 / 6 models tested). | | | | | | | | | | | | |

| **Table S5.2 Neuroticism Facets, Internalizing Symptoms, and Thought Content Predicting *Daily Negative Affect*** | | | | | | | | | | | | |  |
| --- | --- | --- | --- | --- | --- | --- | --- | --- | --- | --- | --- | --- | --- |
|  | **N1 Anxiety** | | **N2 Angry Hostility** | | **N3 Depression** | | **N4 Self-consciousness** | | **N5 Impulsivity** | | **N6 Vulnerability** | |  |
| *Predictors* | *Estimates* | *p* | *Estimates* | *p* | *Estimates* | *p* | *Estimates* | *p* | *Estimates* | *p* | *Estimates* | *p* |  |
| **Level 1 (Within-person)** | | | | | | | | | | | | |  |
| Internal-past TC | **0.06 (0.02 – 0.10)** | **0.001** | **0.05 (0.04 – 0.06)** | **<0.001** | 0.05 (0.01 – 0.09) | **0.011** | **0.05 (0.04 – 0.06)** | **<0.001** | **0.05 (0.02 – 0.08)** | **0.001** | **0.09 (0.06 – 0.12)** | **<0.001** |  |
| External-present TC | -0.00 (-0.13 – 0.13) | 0.971 | -0.01 (-0.04 – 0.02) | 0.604 | 0.05 (-0.06 – 0.17) | 0.362 | -0.01 (-0.04 – 0.02) | 0.596 | -0.02 (-0.12 – 0.08) | 0.709 | 0.12 (0.02 – 0.23) | **0.022** |  |
| **Level 2 (Between-person)** | | | | | | | | | | | | |  |
| Internal-past TC | **0.07 (0.04 – 0.09)** | **<0.001** | **0.06 (0.04 – 0.08)** | **<0.001** | **0.07 (0.04 – 0.09)** | **<.001** | **0.07 (0.04 – 0.09)** | **<0.001** | **0.07 (0.04 – 0.09)** | **<0.001** | **0.06 (0.04 – 0.09)** | **<0.001** |  |
| External-present TC | 0.03 (-0.02 – 0.07) | 0.249 | 0.03 (-0.02 – 0.07) | 0.238 | 0.02 (-0.02 – 0.06) | 0.410 | 0.02 (-0.02 – 0.06) | 0.363 | 0.02 (-0.03 – 0.06) | 0.458 | 0.02 (-0.02 – 0.07) | 0.295 |  |
| Neuroticism facet | **0.07 (0.03 – 0.11)** | **<0.001** | **0.33 (0.10 – 0.55)** | **0.005** | **0.09 (0.05 – 0.13)** | **<.001** | 0.31 (0.04 – 0.57) | **0.022** | 0.04 (0.01 – 0.08) | **0.018** | **0.06 (0.02 – 0.11)** | **0.003** |  |
| Internalizing symptoms | 0.27 (-0.01 – 0.56) | 0.059 | **0.50 (0.28 – 0.73)** | **<0.001** | 0.01 (-0.32 – 0.34) | 0.945 | **0.44 (0.17 – 0.72)** | **0.001** | **0.54 (0.31 – 0.76)** | **<0.001** | **0.38 (0.11 – 0.65)** | **0.006** |  |
| **Cross-level interactions** | | | | | | | | | | | | |  |
| Facet x IP within | -0.00 (-0.00 – 0.00) | 0.522 | 0.00 (-0.01 – 0.01) | 0.941 | 0.00 (-0.00 – 0.00) | 0.914 | -0.00 (-0.01 – 0.01) | 0.756 | -0.00 (-0.00 – 0.00) | 0.834 | -0.00 (-0.00 – -0.00) | **0.023** |  |
| Facet x EP within | -0.00 (-0.01 – 0.01) | 0.938 | -0.01 (-0.04 – 0.02) | 0.573 | -0.00 (-0.01 – 0.00) | 0.279 | -0.01 (-0.05 – 0.03) | 0.699 | 0.00 (-0.00 – 0.01) | 0.827 | -0.01 (-0.02 – -0.00) | **0.012** |  |
| Int. x IP within | 0.01 (-0.00 – 0.02) | 0.154 | 0.01 (-0.00 – 0.02) | 0.236 | 0.01 (-0.01 – 0.02) | 0.505 | 0.01 (-0.00 – 0.02) | 0.207 | 0.01 (-0.00 – 0.02) | 0.193 | **0.02 (0.01 – 0.03)** | **0.005** |  |
| Int x EP within | 0.00 (-0.04 – 0.04) | 0.940 | 0.01 (-0.03 – 0.04) | 0.762 | 0.02 (-0.03 – 0.07) | 0.372 | 0.01 (-0.03 – 0.05) | 0.769 | -0.00 (-0.03 – 0.03) | 0.958 | **0.04 (0.00 – 0.08)** | **0.042** |  |
| *Note*: Bold indicates *p* < .008 (.05 / 6 models tested). | | | | | | | | | | | | | |
